# Co-creation: a collaborative odyssey in dental education with students at the helm

**DOI:** 10.1038/s41415-024-7849-y

**Published:** 2024-09-27

**Authors:** Faith Campbell, Helen Rogers

**Affiliations:** 41415243746001 School of Dentistry, University of Dundee, UK; 41415243746002 School of Dental Sciences, Faculty of Medical Sciences, Newcastle University, UK

## Abstract

Co-creation may be described as collaborative innovation towards a shared goal. It is increasingly being applied in education to develop interventions to support the development of various aspects of educational programmes, including dental education. Students are valuable partners in the process and their unique perspective allows for relevant and novel curricular developments. Other stakeholders within an institution, such as educators, subject experts and programme leads, are also frequently involved. The co-creation process has been reported to be mutually beneficial for all parties.

Benefits of co-creation for students include the development of personal and professional skills that are not conventionally taught within a curriculum. Staff can feel more inspired and engaged. The process can lead to more inclusive and socially relevant curricula. There are also associated challenges, such as gaining adequate support and buy-in from stakeholders to ensure success. This paper explores the concept of co-creation and its application in education, providing recommendations on how it may be successfully applied within the context of dental education.

## Key points

- Describes co-creation, a novel method for developing education.
- Describes the benefits and challenges of co-creation.
- Provides evidence and experience-based guidance for implementing co-creation for readers.

## Introduction

### What is co-creation?

Co-creation is a ‘reciprocal process through which all participants have the opportunity to contribute equally, although not necessarily in the same ways, to curricular or pedagogical conceptualisation, decision-making, implementation, investigation, or analysis'.^1^ Co-creation is also referred to in the literature as students as partners, though we will use the term co-creation as this encompasses collaborative innovation toward a shared goal.

Co-creation is novel in higher education and is increasingly being used as a method for developing interventions to improve many aspects of educational programmes. This ranges from standalone educational resources or activities that enhance the curriculum to entire units or curricula. This methodology can provide opportunities for dental education when considering relevant and meaningful developments within programmes of study.

This paper will discuss the concept of co-creation, examples of successful application of co-creation as a research and curricular development methodology, and practical recommendations for implementing co-creation within the context of dental education.

### Theory underpinning co-creation in education

Although undergraduate students are not yet dentists, they are experts at being dental students and through this ‘expert student' lens, they can make a significant contribution to improving higher educational practice.^2^ Similarly, students are in an excellent position to provide feedback and ideas for innovation, when given the correct platform.^3^ This perspective is described as the ‘student voice', based on the theory that students have a unique perspective on the educational process that they are participating in.^4^^,^^5^ Students should be able to communicate this with, be listened to by, and expect a response from, their educators.^4^^,^^5^

There are an array of benefits, and some challenges, associated with co-creation, outlined in Table 1.

**Table 1:** Summary of benefits and challenges associated with co-creation in higher education

For students	For staff	For institutions
**Benefits of co-creation**		
Engagement in the research process, increased motivation and ownership for learning, and increased sense of confidence and self-awareness, alongside a sense of belonging to university, discipline and community^6^ Deeper learning in the subject area^8^^,^^11^ Greater sense of agency over education^1^ Develop skills that increase employability^1^ Improved academic performance and quality of work^9^ A more transparent learning process^9^ Opportunity to incorporate identity and beliefs in the learning process^11^	May feel more inspired regarding education^1^ Clarify sense of self as an educator^1^ Develop their understanding of learning and teaching by reconceptualising it as a collaborative process^1^	More ‘inclusive and relevant' courses^10^ Improved course outcomes and attendance^10^ Better curricular design^10^ A more transparent learning process^9^ Business-related benefits, such as helping with university branding and attracting future students^7^ Improved student retention and ‘loyalty'^3^^,^^12^
More socially relevant curriculum^9^ Shift of student focus from grading to learning^9^		
**Challenges associated with co-creation**		
Power imbalance, the inability to completely address systematic inequalities and often, only a small number of students and staff are involved^13^ Lack of process and content expertise^28^ Voice fatigue and the perception that their concerns go unheard^28^	A lack of engagement, or even hostility and apathy from some academics towards co-creation^29^ A lack of wider faculty/institutional support^15^ Giving up control and even a feeling of insecurity^28^ Perceived threats in opening up for change, scepticism on the process and the need to change style of communication with students^28^	Future translation of the research^15^ Risk of blindly accepting student opinions without critical thinking from those experienced in teaching^11^ Lack of support and recognition the value of co-creation^19^
Challenge of building a trusting relationship, shifting mindset around traditional hierarchical roles and uni-disciplinary perspectives^15^		
	Practical issues of logistics, management of timelines and time commitments for success^15^ An increased initial time investment which is often more than repaid when students are able to take a more active role in the learning process^11^ Approaches that are disingenuous and leave students as ‘outsiders' to the process risk alienating them^18^	

### Applications of co-creation

While the number of studies describing co-creation is increasing, there is still a paucity in the evidence available to inform best practice approaches to implementation. A recent systematic review of co-creation in higher education found this to be an area that requires further research to enable evaluation of the approach and design of future approaches based on this knowledge.^7^

Co-creation has been used to develop workshops, podcasts and short videos; refine unit guides; and design learning content, the learning environment, and web-based solutions to address educational problems.^7^ Co-creation between staff and students has been used to successfully design relevant curricula in the arts at the University of Edinburgh and also in the USA and Ireland.^11^ Students have been involved as co-researchers to provide feedback on the development of elective study programmes within Glasgow Dental School to inform further refinement of this aspect of the course, which might also be viewed as an aspect of co-creation.^14^

The co-creation process will be unique to each situation and setting. It is important that the approach is adapted to suit the individuals within that setting to ensure that it is functional and successful with consideration to the skillset of individuals, the goal of the project and the opportunities and limitations provided by that working environment. Examples of this include the availability of time and resources and whether existing relationships and programmes of co-creation are available for support.

## Case studies in dental education

Co-creation is increasingly being applied within dental education and there is currently a community of practice within the Association for Dental Education in Europe to steer research in the emerging field.^14^^,^^15^^,^^16^^,^^17^ There are increasing numbers of publications on co-creation in higher education.^7^ A recent systematic review of co-creation in higher education worldwide included 128 papers and found that the most common reason (35%) for the application of co-creation was in ‘educational programme design'.^7^

While co-creation is scarcely reported within higher education literature, it is even less common within dental education. We will discuss two case studies of recent successful applications within dental education to illustrate the process.

### Case study 1

At the University of Sheffield, co-creation has been applied to develop a comprehensive intervention to facilitate deep reflection on clinical practice in paediatric dentistry, following a study that found that using a clinical logbook alone to do so was ineffective in this setting.^19^ This project involved five undergraduate dental students who had participated in a previous study on reflective practice, building on existing collaborations in research, described as a successful approach to developing a co-creation team.^11^

A core co-creation team of five students and three researchers was developed within a wider research team to enable open and clear communication between students and researchers without risking diluting the student voice. Students received recognition for their involvement which included formal Higher Education Achievement Report (HEAR) recognition within the institution. Student co-creators also led in the dissemination of both research findings and their perspectives on co-creation at local and national conferences and their deepened knowledge of reflection through publication.^20^^,^^21^

The impact of this work was the successful development of an intervention to facilitate deep reflection, which included the creation of an online safe space to reflect on clinical activity at a time chosen by students, and educational resources for students and educators regarding reflective practice. Both were led by students.

The intervention developed to address the deficiency in the curriculum was stronger due to being developed for students by students, and the case for acceptance and integration of the change is more convincing.

### Case study 2

At Newcastle University, co-creation has been used to address deficiencies in teaching in cultural competence, which were highlighted through a qualitative study of undergraduate dental and hygiene and therapy students (publication forthcoming).^22^ Students reported struggling when delivering dietary advice to children and families from different cultural backgrounds and were unsure how to analyse the sugar content of food and drink that they were unfamiliar with. Further review revealed a lack of appropriate learning resources to support students in developing their cultural competence in this area.

Five student co-creators were employed and tasked with developing a series of sustainable educational resources to improve cultural competence in dietary advice delivery (publication forthcoming).^22^ They led a series of focus groups with students from different geographical areas, which they selected to reflect the multicultural population local to their university. Through these focus groups, the co-creators gained insight into the food and drink typically consumed by these communities and used recipes to analyse the sugar content of these. They subsequently produced a series of sustainable educational resources to teach their fellow students about the culture, traditions and typical food and drink consumed within the area that they were focused on. The co-creators also produced a diet dictionary of typical food and drink from each geographical area and culture, alongside the respective sugar content, for easy reference in a clinical setting.

The educational resources have been embedded within the undergraduate curriculum for dentistry and hygiene and therapy students. The diet dictionary has been printed and laminated and is available on all undergraduate clinics for students to refer to when delivering dietary advice.

## Student perspectives on co-creation

Students and researchers in both case studies found the use of co-creation enjoyable and effective.^22^^,^^23^ Students reported that recognition through HEAR and involvement in dissemination, and the recognition of the value of their voice in developing a change in the curriculum, has been a positive experience, both educationally and personally.^22^^,^^23^

## Practical steps to undertake co-creation

Recommended steps for undertaking co-creation are outlined in Table 2 based on author experience and available literature.

**Table 2:** Practical steps for undertaking co-creation

Details	Rationale
**Step 1: Formation of the co-creation team**	
Establish clear goals for the project	It is important that this is clarified with learners and stakeholders from the outset^28^
Recruitment: this should be open to all students and invitations shared in an accessible manner, for example, emails to all eligible participants	It is ideal to have an equal number of researchers and students to avoid power imbalance^29^
Provide information regarding time commitments, approximate deadlines and requirements of the project before committing to participating	Clear communication of this is described as essential for success in the qualitative research synthesis of co-creation in higher education^15^
Involve other stakeholders within the institution, who may be affected by the project, such as theme leads, heads of year and pastoral teams	Stakeholders with significant involvement and interest in the developed intervention and could benefit from involvement in the co-creation process^15^
**Step 2: Definition of roles, responsibilities and commitments**	
Student roles: Meet deadlines, maintain communication with the team and attend meetings when required. It may be helpful to confirm the format of these initially to ensure they are mutually agreeable Contribute their perspective to the project, including but not limited to, proposed design, development and feedback on interventions, participation in implementation, evaluation, and possible dissemination of research findings	The involvement of student voices and contributions as equals in publication assists in the endeavour for a more egalitarian approach to education^6^
Researcher roles: Arrange formal academic support for the student co-creators Ensure that the co-created design is appropriate and aligned with regulatory and university standards for undergraduate education. This may include developing briefs and reviewing works for the students Act as an intermediary between the co-creators and faculty	To support open, bidirectional feedback on the process and outcomes, connecting teachers and learners throughout^28^
**Step 3: Preparation and support for student co-creators**	
Provision of independent guidance for students for the project duration from teams experienced in student involvement in teaching and learning, for example, academic skills or teaching and learning teams external to the dental school	Such arrangements can help in providing individual support and discussing experiences and best practices in contributing to co-creation. Independent, hands-on support from faculty developers has been described as essential for success^28^
Arrange recognition for student contribution, discuss this with them and ensure that it is meaningful for them. This may be in academic recognition within the institution, formal recognition through an institutional extra-curricular skills scheme or financial recognition of support	Recognition of efforts in co-creation, alongside dissemination and sharing of results, for both learners and educators, helps in establishing a culture of empowerment for sustainable implementation of co-creation^28^
Prepare students by providing teaching the theme of the intervention, for example, reflective practice, dental anatomy or professionalism. This can be done through preparatory sessions with the external supporting team. It is suggested that this is initially done without researchers being present to allow for the students to feel open in their learning	This allows development knowledge and reflective skills to empower students to meaningfully contribute to the project and ensures that students are not working far beyond their expertise^28^
Communicate with students through the most appropriate means for each task. This may be face to face, virtual meetings, or establishment of a group chat such as a Microsoft Teams	Recommended to afford co-creators more control and choice over how they share their thoughts.^28^ Less traditional communication, for example through Google Jamboard (Google, LLC) can empower and spark creativity for student co-creators and the use of the digital communication can break down hierarchical barriers^28^
**Step 4: Planning and development of intervention**	
Students to brainstorm suggestions for interventions as a group on their own (with or without support from external team) and discuss these with research team	This helps to facilitate student initiative and enables them to take ownership of original ideas
Research team to review the findings of the brainstorming to discuss the feasibility of student suggestions and explore the development of a suitable proposal	Suggest that this is undertaken without students being present to not over-burden them and to enable open discussion on the ideas from an academic perspective
Intervention reviewed by core co-creation team and wider stakeholders within the dental school	
Regular discussions with other stakeholders and the core researchers and negotiation of implementation of intervention. Key themes encountered in negotiation may be time and resource requirements of proposal information governance and ensuring compliance with regulatory body requirements	To consider the practicalities, feasibility and acceptability of the proposed intervention that may not have been anticipated by core research team members in the planning stage. Students should not be involved in the negotiation on the implementation of the intervention to avoid any power imbalance
Some practical aspects of development may often need to be undertaken by experts in that field, for example those with skills in teaching or IT. Students should be given the opportunity to review every aspect of this and contribute to the development where appropriate	This offers students the opportunity to develop further skills and expertise from working with experts. It is important to actively engage student co-creators in every stage of the project
**Step 5: Implementation of intervention**	
Students should be given the opportunity to share the developed intervention with peers, staff and their institution. They should also be involved in dissemination of their work, including skills developed and their experience, within the institution and with the wider research community. Opportunities for student involvement in evaluation of intervention should also be provided	This recognises their contribution and gives more power to the intervention^19^

## Other partners in co-creation

As discussed in Table 1, higher education institutions comprise a range of stakeholders that could be involved when planning and undertaking a co-creation project. It is essential to ensure that all stakeholders who will be impacted by the project outcome are included as partners in the development so that they feel valued and can provide feedback, rather than feeling blindsided by, or excluded from, an intervention. This approach can also help in foreseeing potential issues that students or educators may not anticipate, hence increasing the chance of the final product being successful. For example, to change assessment methods, it would be pertinent to include and consult examiners and administrative staff, or, for a novel computerised development, those with the computer technology skills to construct the new tool should be involved.

Other partners to consider for involvement include those from communities local to the higher education institution, international partners, and patients with lived experience of receiving oral healthcare and accessing healthcare services. While the authors are not aware of any of these approaches to partner inclusion being mentioned within the dental co-creation literature, anecdotally, some of these approaches have been used informally in curricular development. Examples of these approaches being used successfully would be a welcome contribution to the evidence base in co-creation.

As the regulatory body for dentists and dental education, the General Dental Council (GDC) is also a potential stakeholder for co-creation within dental education. The authors would not recommend that they are directly involved in the process of curricular design, as they are not prescriptive on the teaching methods for undergraduate dentistry.^24^ However, they do appreciate that the GDC should be included to an extent as a wider partner. In current work to develop a national core curriculum for paediatric dentistry, the GDC will play a key role in contributing to, and ultimately endorsing, any national curricular being developed.^25^^,^^26^ This approach to partner inclusion will be essential to ensure widespread adoption of any new national curriculum.

The authors would recommend that all members of co-creation teams working in undergraduate dental education are aware of the standards for education documents and the relevant requirements from the GDC to ensure that developments are compliant.^24^ Within professionally regulated courses, such as dental surgery and dental therapy, the content of the courses is prescribed by the GDC.^24^ As such, the opportunity for co-creation with students in developing the content of a curriculum ‘ground up' is limited. However, there are a wealth of areas in which students can be involved as partners, such as in the implementation of the curriculum, resource development and course evaluation.

The following key recommendations should be applicable to co-creation, whatever the scenario:

1. Clear roles and responsibilities with formal support for student co-creators - it is useful to define roles and responsibilities for the benefit of all parties.^19^ It is also helpful to position students as peers with valuable perspectives which helps to break down hierarchal barriers and build relationships.^27^ Empowering students with the support in developing knowledge on both the topic area and skills in co-creation enables them to participate fully^28^
2. Meaningful reward and recognition - to ensure involvement in co-creation for dental education is viewed as meaningful for students, we would advise asking students how they would like to be thanked for their time in participation. This ensures that student contribution is not viewed as a tokenistic or transactional. Examples of meaningful ways that students have been recognised include formal recognition through university programmes, such as the HEAR, used at the University of Sheffield, to demonstrate skills development to future employers.^19^ Other approaches include formal employment and opportunities to be involved in dissemination of research through student-led publication and presentation^19^
3. Flexibility of all partners - co-creation is an iterative process and therefore by not allowing flexibility in deadlines and adapting to change, one would set themselves up for failure
4. Establishment of institutional engagement - this is essential for success.^28^ A lack of institutional support is also frequently reported as a barrier for the process.^19^^,^^29^ This may look like funding, time and space for co-creation, alongside allowing risk-taking and the change of hierarchal roles that are part of the process and supporting initiatives that result from it^28^
5. Consider the sustainability of the intervention - sustainability of co-creation has been described as a challenge.^15^^,^^19^ When student co-creators who have championed innovation have inevitably moved on, there is a risk that their intervention falls to the wayside and institutions revert to ‘what they have always done'. Ensuring that interventions are sustainable, for example, educational resources that can be reused, or integrating the change into curriculum, is reported to increase the success of any intervention.^15^^,^^30^

## Conclusion

Co-creation is recommended as an effective methodology for developing interventions and curricular change within dental education. There is a growing and compelling body of evidence supporting this and the authors would encourage the involvement of students as co-creators in dental education research.

## Data Availability

Data sharing is not applicable to this article as no new data were created or analysed in this study.
